# Ascomycota: Diversity, Taxonomy and Phylogeny, 2nd Edition: Editorial

**DOI:** 10.3390/jof11060419

**Published:** 2025-05-30

**Authors:** Asha J. Dissanayake, Jian-Kui Liu

**Affiliations:** School of Life Science and Technology, Center for Informational Biology, University of Electronic Science and Technology of China, Chengdu 611731, China; asha.janadaree@yahoo.com

## 1. Introduction

It’s crucial to emphasize a universal conservation of all life on the earth, including not only plants and animals but also fungi. The fungal kingdom is thought to encompass between 2.2 and 3.8 million species, with 2–3 million entities yet to be exposed [1,2,3]. The number of fungi has been the subject of numerous recent review articles, consistently proving to be an interesting topic. Estimates of the number of fungal species have changed in the past decade due to the revolutionary impact of new DNA sequencing technology on studies of fungal taxonomy and diversity.

The most diverse phylum of fungi is Ascomycota. The spores of Ascomycota are easily dispersed by wind, water, soil, or insects, and they are well suited to endophytic, saprobic, parasitic, or mutualistic life modes [4]. They may reproduce both asexually and sexually. Taxonomists, plant pathologists, chemists, ecologists, and landowners must be able to accurately identify, name, and discuss the Ascomycota species they come into contact with on a daily basis in order to preserve biodiversity. Different species ideas and delimitation techniques have been widely employed in recent decades to identify novel species, establish species boundaries, and investigate connections among closely related species [5,6].

Fungal plant pathogens potentially incite devastating ecological and economic damage to agriculture and forestry and can also cause severe damage to natural ecosystems [7,8]. Classification of plant pathogens is not only crucial for identification, but also carries information regarding their diversity and potential functions. However, given the huge number of broad and narrow species concepts and multiple synonyms with unclear typification, plant pathologists and taxonomists are often confronted with ambiguity for newly identified pathogens. Many known fungal species need to be recollected and epi- or neotypified in order to secure the application of old names already in use and resolve their DNA phylogeny. Subsequent to the end of the long-standing dual nomenclature for fungi [9,10] and the connection of different morphs to a single name [11], it’s clear that the platforms are required to ensure that these data could be easily and effectively published.

It has become clear that there are several unidentified Ascomycotal species and new host or geographical records for which there is no scientific repository, similar to the previous special issue of “Ascomycota: Diversity, Taxonomy and Phylogeny, 1st Edition”. Science would likely never be able to end-up with describing or categorizing the majority of them. Hence, the new series on “Ascomycota: Diversity, Taxonomy and Phylogeny, 2nd Edition” was proposed to allow mycologists a compelling platform to publish new genera, new species, or to emphasize the relevance of significant fungi. This special issue introduced a new series of research articles that will expand the existing knowledge of fungal biodiversity and fungal numbers of Dothideomycetes, Sordariomycetes and Arthoniomycetes. Additionally, this will divulge the importance of pathogenic fungi, entomopathogenic fungi, as well as the diversity of lignicolous freshwater fungi.

## 2. A Synopsis of the Key Papers in This Special Issue

### 2.1. Introduction of New Ascomycota Species

This offers a prompt and simplified series for researchers to introduce new fungal species in Ascomycota and moreover to emphasize the environments where these fungi were isolated. This special issue will provide opportunities to merge morphological observations with DNA sequence data, providing a means for rapid and accurate species identification.

#### 2.1.1. Introduction of New Dothideomycetes Species

Dothideomycetes were previously known as Loculoascomycetes and is the largest class of ascomycetes [12,13]. Hyde et al. [12] confirmed that Dothideomycetes comprises two subclasses viz. Dothideomycetidae and Pleosporomycetidae, while other uncertain orders and families were treated as incertae sedis in Dothideomycetes. Hongsanan et al. [13] accepted three orders with 25 families in Dothideomycetidae while four orders with 94 families in Pleosporomycetidae.

**Additions to Dictyosporiaceae: *Neoxylochrysis typhicola* comb. et gen. nov., Two New Species and Four New Host Records from Medicinal Plants in Southwestern China [Contribution 1]**

The Dictyosporiaceae species investigated in this study were collected from ten different medicinal plants in Guizhou and Sichuan Provinces in China. Medicinal plants serve as vital resources for preventing and treating diseases. Multi-locus phylogenetic analysis based on combined ITS, LSU, SSU and *tef1-α* datasets revealed a novel genus, *Neoxylochrysis*, which accommodates *Neoxylochrysis typhicola* comb. nov. (≡*Pseudocoleophoma typhicola*) and two new species (*Dictyocheirospora alangii* and *Pseudocoleophoma rosae*). In addition, four new host records are introduced, and all of the taxa identified in this study were saprophytic fungi.

**Phylogenetic and Morphological Analyses Reveal Twelve New Species of the Genus *Patellaria* (Dothideomycetes, Ascomycota) from Mexico [Contribution 2]**

García-Jacobo et al. described 12 new *Patellaria* species in Mexico supported by morphological and molecular data (ITS-LSU-SSU). *Patellaria* species are saprobes that grow on decomposing wood, stems, or bark and are found in both terrestrial and marine environments. Previous studies have identified only five species of *Patellaria* using morphological and molecular data, since the majority of species have been characterized based on morphological traits, such as the size and number of septa in the ascospores, the size and form of the asci and the shape and color of the ascomata.

**New Species of *Byssosphaeria* (Melanommataceae, Pleosporales) from the Mexican Tropical Montane Cloud Forest [Contribution 3]**

*Byssosphaeria* species are saprobes, endophytes, and parasites of woody angiosperms. *Byssosphaeria* is a monophyletic genus of the family Melanommataceae and is composed of 29 species. Cobos-Villagrán et al. described four saprobic *Byssosphaeria* species (*B. bautistae*, *B. chrysostoma*, *B. neorhodomphala*, and *B. neoschiedermayriana*) in mountainous mesophilic forests from the states of Hidalgo, Puebla, and Oaxaca in Mexico through morphological characteristics and the phylogenetic analysis of molecular markers (ITS, SSU, LSU, *tef1-α*).

#### 2.1.2. Introduction of New Sordariomycetes Species

Sordariomycetes is the second largest class of Ascomycota [3] having a cosmopolitan distribution and can be found in almost all ecosystems [14]. The classification of the Sordariomycetes has changed drastically over the past decade [3]. The six subclasses of Sordariomycetes are Diaporthomycetidae, Hypocreomycetidae, Lulworthiomycetidae, Savoryellomycetidae, Sordariomycetidae and Xylariomycetidae [15,16,17].

**Morpho-Molecular Characterization Reveals a New Genus, Three Novel Species and Two New Host Records in Xylariomycetidae [Contribution 4]**

Xylariomycetidae is a phylogenetically and morphologically diverse fungal assemblage containing three orders, viz., Amphisphaeriales, Delonicicolales and Xylariales. An investigation of the diversity of microfungi on oil tree plantations in Sichuan Province by Li et al. described a few taxa of Xylariomycetidae, including a novel anthostomella-like genus, *Bicellulospora* (*B. elaeidis*) and two new species of *Amphisphaeria*, namely *A. oleae* and *A. verniciae*, based on multi-gene phylogenetic analyses (ITS, LSU, *rpb2* and *tub2*) coupled with morphological characteristics. They have reported two new host records of *Amphisphaeria micheliae* and *Endocalyx ptychospermatis* on woody oil plants.

**Taxonomy and Phylogeny of Eight New *Acrophialophora* Species (Sordariales, Chaetomiaceae) from China [Contribution 5]**

*Acrophialophora* is a thermotolerant genus that belongs to Chaetomiaceae. Incorporating morphological characteristics and multi-locus phylogenetic analysis (ITS, LSU, *tub2* and *rpb2*), Peng et al. described eight new species of *Acrophialophora* (*A. curvata*, *A. fujianensis*, *A. guangdongensis*, *A. longicatenata*, *A. minuta*, *A. multiforma*, *A. rhombica*, and *A. yunnanensis*) from soil samples in China.

**Six Species of *Phyllachora* with Three New Taxa on Grass from Sichuan Province, China [Contribution 6]**

*Phyllachora* is a group of biotrophic, plant-parasitic fungi with high host specificity and a global distribution and is known to cause tar spots on plants. Three new *Phyllachora* species (*P. chongzhouensis*, *P. neidongensis*, and *P. huiliensis*) have been isolated from Poaceae hosts in Sichuan, China. Sun et al. re-examined the original articles of *P. chloridis*, *P. graminis*, and *P. miscanthi*, as they were from Chinese or abbreviated references and were redescribed based on ITS, LSU, and SSU sequence data.

**Three New Species of *Fusicolla* (Hypocreales) from China [Contribution 7]**

Zeng and Zhuang introduced three novel species of *Fusicolla* (*F. aeria*, *F. coralloidea* and *F. filiformis*) from Henan, Hubei and Jiangsu Provinces in China based on morphological characteristics and DNA sequence analyses of the combined *acl1*, ITS, LSU, *rpb2* and *tub2* regions. Studies have shown that members of *Fusicolla* are economically important in the fields of fermentation, ecology, agriculture and human health.

#### 2.1.3. Introduction of New Arthoniomycetes Species

**The Phylogeny and Taxonomy of *Cryptothecia* (Arthoniaceae, Ascomycota) and *Myriostigma* (Arthoniaceae, Ascomycota), including Three New Species and Two New Records from China [Contribution 8]**

In tropical to subtropical woody hosts, *Cryptothecia* and *Myriostigma* are crucial components of crustose lichen communities. As not much is known about these two taxa in China, Xue et al. introduced five species of *Cryptothecia* and *Myriostigma*, including three new species (*M. flavescens*, *M. hainana* and *M. laxipunctata*) and two new records (*C. bartlettii* and *C. inexspectata*) based on SSU, *rpb2* and LSU sequence data.

### 2.2. Plant Diseases Caused by Ascomycota Fungi

The tripartite interaction of host, pathogen, and environment leads to the development of plant diseases. For early pathogen detection, disease prevention, and management, it is essential to accurately identify and comprehend these aspects [18]. Depending on the fungal species, different control and management strategies may be used. Hence, it is imperative that each of these species be accurately identified and given the appropriate name.

#### 2.2.1. Pathogenic Ascomycota Fungi

**Microfungi Associated with Peach Branch Diseases in China [Contribution 9]**

Though Peach (*Prunus persica* L.) is one of the most important and oldest stone fruits grown in China, little is known about the biodiversity of microfungi associated with peach branch diseases. Fungal infections causing peach branch diseases were collected by Zou et al. from a range of peach-growing regions in China. Based on morphology and phylogenetic analysis, the majority of isolates were identified as members of the Botryosphaeriaceae, which includes species of *Botryosphaeria*, *Diplodia*, *Neofusicoccum*, *Phaeobotryon*, and *Lasiodiplodia*. *Ascochyta*, *Didymella*, and *Nothophoma* species of Didymellaceae were also identified. Two new species, *Ascochyta prunus* and *Lasiodiplodia pruni* were introduced.

#### 2.2.2. Entomopathogenic Ascomycota Fungi

**Morphological and Phylogenetic Analyses Reveal Three New Species of Entomopathogenic Fungi Belonging to Clavicipitaceae (Hypocreales, Ascomycota) [Contribution 10]**

Entomopathogenic fungi are widely distributed in China. Whiteflies (Aleyrodidae, Hemiptera) and scale insects (Coccidae and Lecaniidae, Hemiptera) are parasitized by species of *Conoideocrella* and *Moelleriella*. Wang et al. conducted a phylogenetic analysis of combined-gene LSU, *tef1-α*, and *rpb1* to reveal two new species, *Moelleriella longzhuensis* sp. nov. and *Moelleriella jinuoana* sp. nov., in the genus *Moelleriella*, as well as two new records of *Conoideocrella tenuis* and *Conoideocrella fenshuilingensis* in the genus *Conoideocrella*. Wang et al. addressed the calculation of genetic distances for the three genes, showing that the interspecific genetic distances between the new species in this study and other species of the genus were greater than the intraspecific genetic distances.

### 2.3. Aquatic Ascomycota Fungi

Lignicolous freshwater fungi can decompose lignocellulose substrates and release energy and nutrients into the water environment, playing an important role in the freshwater environment [19]. Though they represent a highly diverse group, only around 3900 species are identified from freshwater environments. It shows the significance of continued exploration and suggests that vast freshwater ascomycetes remain undiscovered [20,21].

**Diversity of Lignicolous Freshwater Fungi from Yuanjiang River in Yunnan (China), with the Description of Four New Species [Contribution 11]**

One of Yunnan Province’s six main water systems, the Yuanjiang River (also known as the Red River), originates in the western part of the province. Zhang et al. investigated lignicolous freshwater fungi grow on submerged woody debris in Yuanjiang River and nine fungal species were isolated. Four species—*Aquadictyospora aquatica*, *Dictyosporium fluminicola*, *Myrmecridium submersum*, and *Neomyrmecridium fusiforme*—are identified as novel species based on morphology and multigene phylogenetic analysis. The variety of lignicolous freshwater fungi in southwest China is better understood during this study.

## 3. Conclusions

We appreciate the outstanding work of the distinguished mycologists who have edited this Special Issue, and editors who have made significant contributions to this discipline over the years. We are grateful to the Journal of Fungi, a renowned and widely acknowledged mycology journal, for proposing this Special Issue and that its staff worked so hard to make it happen. We are grateful to all of the exceptional writers for their wonderful contributions.
